# Temporal Sensory Perceptions of Sugar-Reduced 3D Printed Chocolates

**DOI:** 10.3390/foods10092082

**Published:** 2021-09-03

**Authors:** Khemiga Khemacheevakul, John Wolodko, Ha Nguyen, Wendy Wismer

**Affiliations:** 1 Department of Agricultural, Food and Nutritional Science, University of Alberta, Edmonton, AB T6G 2P5, Canada; khemache@ualberta.ca (K.K.); jwolodko@ualberta.ca (J.W.); 2 Chemical and Food Engineering, Ho Chi Minh City University of Technology (HUTECH), Ho Chi Minh City 70000, Vietnam; ntt.ha84@hutech.edu.vn

**Keywords:** 3D printing, chocolate, temporal sensory methods, sugar reduction, TDS, spatial distribution, liking

## Abstract

Sugar-reduced chocolates with desirable sensory qualities and sweetness can be created using a 3D printer by layering chocolates with different sugar concentrations. This study aimed to evaluate the temporal sensory profile, perceived sweetness intensity, and acceptance of prototype sugar-reduced and non-sugar-reduced 3D printed chocolates. A consumer panel (*n* = 72) evaluated the sensory profiles of six-layered chocolates. Sensory profiles were determined by temporal dominance of sensations (TDS), overall sweetness by a five-point intensity scale, overall liking by the nine-point hedonic scale, and differences among chocolates over time were visualized by principal component analysis (PCA). Layering by 3D printing achieved a 19% reduction in sugar without changes in the perceived overall sweetness and overall liking. Layering order of high and low sugar chocolate influenced the perceived overall sweetness and temporal sensory profiles of 3D printed chocolates with different total sugar concentrations. The dominance of attributes associated with milk chocolate was observed to increase sweetness perception while the dominance of attributes associated with dark chocolate was observed to decrease overall sweetness perception. Three-dimensional food printing technology is progressing rapidly, and further sugar reduction could be achieved with refined research methods.

## 1. Introduction

Globally, there is an increasing need for sugar-reduced foods due to the negative impacts of high sugar consumption on human health and higher dietary consumption of total sugars than recommended. Health impacts of a high sugar diet include increased body weight, dental caries, and poor oral health, and the World Health Organization (WHO) recommends less than 10% of total energy intake from free sugars [1]. However, 2015 data revealed that free sugars, added sugars, and total sugars intake contributed to 13.3%, 11.1%, and 21.6% of total daily energy intake of Canadians, respectively [2]. Although total sugar intake declined from 2004 to 2015 for the “sugars, syrups and confectionary” category of food products, they remained among the top sources of total sugars intake for all age groups [3]. Moreover, the “desserts and sweets” group was the greatest contributor of free (57.5%), added (67.3%), and total sugar (41.4%) in the Canadian diet [2]. Considering the importance of reducing sugar in this food category and its popularity, prototype sugar-reduced chocolates were evaluated in this research. 

Arranging different concentrations of tastants in layers within a food structure can alter the sensory profile, thereby allowing a reduction in the tastant without affecting desirable sensory perceptions. This spatial distribution has been demonstrated with sugar in gels [4,5,6], salt in bread [7] and cream-based snacks [8], and fat in gels [9] and sausages [10]. Bread samples with a heterogeneous distribution of salt permitted a reduction of up to 28% overall salt content without compromising saltiness intensity [7]. For sausages with 2% (*w*/*w*) total salt, the sample with an inhomogeneous distribution of salt was liked significantly more than the sausage with a homogeneous distribution, suggesting the method can be used to reduce salt in food without lessening consumer tastant acceptance [10]. Sweetness intensity was enhanced by an inhomogeneous distribution of sugar particles in gels. Of two gelatin gels with 9% sugar (*w*/*w*), a seven-layered sample with an inhomogeneous distribution of sugar particles was perceived to be sweeter than a five-layered homogeneous sample [4]. Of two four-layered agar/gelatin gels, a 10% sugar (*w*/*w*) sample with an inhomogeneous distribution of sucrose and a large concentration gradient of sucrose between the layers, tasted sweeter than a homogeneous sample with 12% (*w*/*w*) sucrose [5]. 

Spatial distribution of sugar can be achieved by 3D food printing (3DFP) using a dual-extruder 3D food printer to create alternating chocolate layers of different sugar concentrations [11]. Three-dimensional food printing is a more accurate and efficient method to create chocolate layers compared to conventional methods using molds and provides the user with the flexibility to create layered designs of different shapes as desired. The research described here studied the effect of a spatial distribution of sugar in three-layered 3D printed chocolates on their sensory attribute profile, perceived sweetness, and overall liking. 

Temporal dominance of sensations (TDS) is a sensory methodology that provides a dynamic view of the evolution of dominant sensory attributes for a food product during the tasting period [12]. Chocolate has a complex sensory profile influenced by its chemical composition and post-harvest processing. Appearance, aroma, flavor, and texture attributes, coupled with a phase change during consumption, contribute to a rich temporal chocolate experience that could influence overall perceived sweetness. A chocolate sensory wheel and lexicon revealed that milk chocolate was most associated with the attributes “creaminess”, “milk/cream” flavor, and “sweet” while dark chocolate was more associated with “hardness,” “snap,” and “bitter” [13]. Therefore, a temporal evaluation of key sensory attributes associated with milk and dark chocolate is valuable to determine chocolate sensory attribute interactions with sweetness perception. 

This research aimed to investigate how layering order of chocolates with different concentrations of sugar would influence the temporal sensory attribute profile, and if changes in the sensory profile influenced perceived sweetness intensity and overall acceptance of sugar-reduced and non-sugar-reduced 3D printed chocolates. It was hypothesized that the sensory profile of chocolates with similar total % sugar would change as a result of the layering, and this was expected to affect overall liking. It was also hypothesized that layering combinations with the high sugar chocolate as the bottom layer would allow chocolate samples with a lower total % sugar to taste just as sweet, if not sweeter, than samples with a higher total % sugar because the tongue has the highest density of taste buds in the oral cavity. 

## 2. Materials and Methods

The plan for this study (Study ID: Pro00097545) was reviewed for its adherence to ethical guidelines and approved by the Research Ethics Board at the University of Alberta. All participants completed written informed consent.

**Figure 1:**
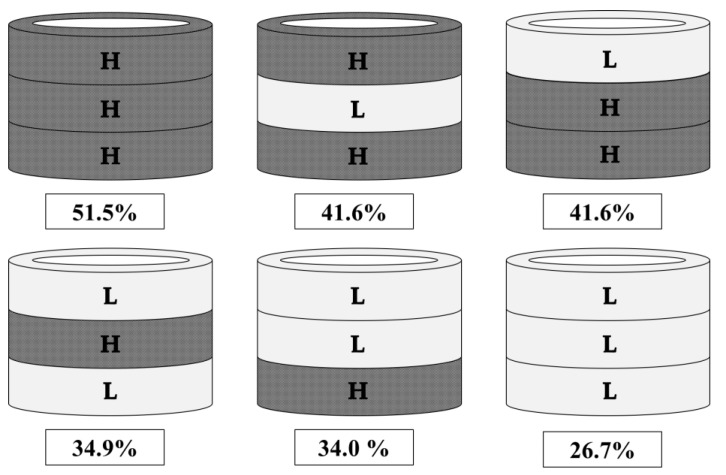
The six three-layered hollow cylinder samples and their total % sugar concentration (*w*/*w*) where H represents a high sugar layer (51.5%) and L a low sugar layer (26.7%). Chocolate samples are named by using upper case letters to represent layering order from bottom to top (HHH, HLH, HHL, LHL, HLL, LLL).

***Chocolate samples*.** Bars of 47% cocoa Swiss dark chocolate (high sugar: H) and 72% cocoa Swiss dark chocolate (low sugar: L) (Western Family ^TM^, Overwaitea Food Group, Vancouver, BC, Canada) were purchased from a local supermarket. Three-layered 3D printed chocolates were created in the shape of a hollow cylinder (diameter: 28.00mm, height: 10.80mm, thickness: 4.37mm). The six samples used for sensory testing had different sugar concentrations and layering combinations from the bottom to the top layer (Figure 1) to generate four % sugar (*w*/*w*) concentrations: 51.5%, 41.6%, 34.0–34.9%, or 26.7%. Two samples were homogeneous (HHH, LLL) and four were inhomogeneous (HLH, HHL, LHL, HLL). The % sugar (*w*/*w*) in HHH (51.5%) is comparable to conventional chocolates sold at grocery stores in North America, therefore, the HHH sample was used in this experiment as the non-sugar-reduced control. 

The samples were manufactured in a food-grade laboratory following the process described elsewhere [14]. Briefly, digital 3D designs were converted into G-code and loaded into the RepetierHost printing software. Then, the chocolates were tempered by heating to 45–55 °C, followed by cooling to 27 °C using the seeding method. Cooled chocolates were fed into the dual extruders of the 3D printer (3Drag 3D Printer with chocolate extruder (Open Electronics, Futura Group Srl, Italy)), and printed at a set temperature of 28 °C (L chocolate) or 32 °C (H chocolate) using an average print speed of 3 mm/s and an average flow rate of 6 mm^3^/s. Samples were prepared in batches for each week of sensory paneling and stored at room temperature (20 °C) for no more than one week prior to sensory evaluation to ensure similar freshness. For maximum freshness, the chocolates were placed into plastic cups with lids, the cups were put into air-tight freezer bags and the bags were placed inside plastic containers which were stored in a dark and dry cupboard.

***Sensory panel.*** Students and staff (*n* = 72) at the University of Alberta, aged 18 and older who liked and were regular consumers of both milk and dark chocolate were recruited as participants for the study through flyers and university email lists. Immediately prior to evaluations participants were trained by the following procedure:

1. Familiarization with the chocolate TDS attributes based on the attribute definition list (Table 1).
2. Introduction to the TDS methodology, sweetness intensity scale, and overall liking scale.
3. Familiarization with the interface in the Compusense^®^ Cloud sensory software (Compusense Inc., Guelph, Ontario, Canada) by performing the sensory evaluation procedure using a square of H chocolate as a warm up sample.

***Sensory evaluation.*** For each sample, participants completed an online questionnaire on Compusense^®^ Cloud sensory software. They were asked to:

1. Complete a TDS evaluation by selecting from a list of eight attributes, the most dominant attribute perceived over 120 s.
2. Indicate their opinion of the sweetness of the sample on a 5-point intensity scale (1 = not at all sweet, 5 = extremely sweet).
3. Rate the sample for overall liking on a 9-point hedonic scale (1 = dislike extremely, 9 = like extremely).
4. Fill out a demographics and product use questionnaire.

TDS attributes to be evaluated by the panel were selected by first reviewing attributes and their definitions used in published chocolate sensory [13] and TDS research [15,16,17,18,19,20,21]. Preliminary testing within the research group determined that eight sensory attributes were appropriate for the samples: sweet, bitter, chocolate flavor, milky flavor, creamy, melting, hard/brittle, and soft. A training procedure, the maximum amount of time needed to complete the evaluation (2 min), and the correct orientation for placing samples into the mouth for evaluations were also trialed by the group. 

The TDS evaluation procedure followed recommended guidelines in International Standards Organization (ISO) 13299:2016(E) [22]. Participants started the evaluation by pressing the “play” button as they placed the sample into their mouth, chewed each sample three times, and let it melt on the tongue for the remainder of the time. Samples were eaten in the orientation presented in the cup, ensuring the bottom layer of the sample was on the tongue. While eating, the attribute that stood out the most was selected from the list provided and re-selected as the strongest attribute changed. An attribute was considered dominant until another attribute was chosen. Participants selected only one attribute at a time but could select an attribute more than once and did not have to select all of the attributes. The “stop” button was pressed when the chocolate sample was gone from the mouth.

***Experimental design.*** Sensory evaluations were conducted at the sensory panel rooms in individual sensory booths under controlled light and air conditions. A balanced incomplete block design for six treatments was used to determine the sample presentation order. Participants were asked to taste and evaluate three of the six samples in one 30 min session. Each sample (approximately 3.3g) was presented in 60mL plastic cups (P200N, Solo^®^ translucent portion containers, Dart Container Corporation, Mason, Michigan, USA) with a plastic lid (PL200N) at room temperature (20 ℃). A 30 s break was provided between each evaluation in order to cleanse the palate with deionized water. 

***Statistical analysis.*** All analyses were performed using R studio (Version 1.1.463, R Studio Inc., 2009–2018). TDS data were analyzed following the procedure described for TDS curves and TDS difference curves computation in Pineau, N. et al., (2009) [23] using the tempR: Temporal Sensory Data Analysis package [24]. The data from each panelist was standardized from X = 0.00 (first selection of dominant attribute) to X = 1.00 (no more attributes selected) to account for differences in duration in the mouth. The dominance rates (proportion of participants that cited an attribute as dominant at each moment in time) were calculated, smoothed, and plotted against standardized time. TDS curves of all attributes were plotted on the same graph for each sample. The chance and significance levels were calculated and indicated by dotted lines on the graph [23]. TDS difference curves were plotted to compare among all products and among products with similar overall perceived sweetness based on the mean perceived sweetness intensity. TDS difference curves were computed by subtracting the dominance rates of two samples for each attribute at each point of time. To highlight the differences between products over the evaluation period, only the significantly different dominance rates were plotted. 

Overall sweetness and liking data were analyzed by one-way Analysis of Variance (ANOVA) with Tukey’s HSD Test (*p* ≤ 0.05) using the nlme [25] and lsmeans [26] packages to investigate differences in perceived sweetness intensity and overall acceptance among samples. Samples were set as a fixed factor and panelists as a random factor. 

Principal component analysis (PCA) was conducted with the tempR package and was used to visualize product differences during the temporal evaluation. Product trajectories were plotted for each 3D printed chocolate using a data frame with attributes tested in the columns and dominance rates over standardized time in rows [27]. Descriptive statistics were used for demographics and consumption habits data. 

## 3. Results

### 3.1. Participants

The majority of participants were female (67%) aged 18–25 (58%) who consumed chocolate once per week or more often (86%) (Table 2). About half of the participants (54%) preferred milk chocolate to dark chocolate.

### 3.2. Overall Sweetness and Liking

All samples were rated as “not very sweet” to “moderately sweet” (2.3–3.3) on the 5-point scale, and three sweetness groups were identified (Table 3). Samples with 41.6% sugar (HLH, HHL) were similar in sweetness to the control sample (HHH) with 51.5% sugar. This suggested that 3D printed sugar-reduced chocolates created by spatial distribution of sugar in layers, with up to 19% reduction in sugar (HLH, HHL) can taste as sweet as a non-sugar-reduced 3D printed chocolate (HHH). On the other hand, samples with 34.0% (HLL) and 34.9% (LHL) were similar in sweetness to the lowest sugar sample (LLL) with 26.7% sugar, but not to the control sample (HHH). This indicated that a sugar reduction of 32% from control was detected by participants. Although LHL was similar to LLL, it was also similar to HLH and HHL which had 19% more sugar. This further supports that a sugar reduction of 19% by spatial distribution in layers in chocolate can be achieved without changes in sweetness perception. 

There were no significant differences in overall liking between the six samples (Table 3). They were all liked slightly to moderately (6.4–6.9).

### 3.3. TDS Curves

TDS curves of the eight sensory attributes were plotted for each 3D printed chocolate sample (Figure S1) to observe the temporal sensory profile of each sample. The chance (0.125) and significance (0.204) levels are indicated as dotted lines on the plots. Attributes were considered to be dominant when the dominance rate exceeded the significance level. The peak dominance rates for all attributes ranged from 0.22–0.47. To observe possible layering effects, the dynamic profile of the chocolates was interpreted by attribute and standardized time quadrants: Q1 (0.00–0.24), Q2 (0.25–0.49), Q3 (0.50–0.74), Q4 (0.75–1.00). 

“Milky flavor” was never dominant in any sample, whereas “Hard/Brittle” was dominant for all samples at the beginning of evaluations in Q1 with peak dominance rate ranging from 0.36 (HHH) to 0.44 (LLL). 

“Chocolate flavor” was also dominant for all samples. Peak dominance rates ranged from 0.33 (HHH) to 0.47 (HLL) and varied in dominance periods. For HHH, chocolate flavor was dominant in intervals during Q1-Q3, while for LLL, it was also dominant in intervals Q1–Q3, especially Q2. 

In samples HLH and HHL, this attribute was dominant in intervals from Q2–Q4. Chocolate flavor was dominant in intervals throughout the evaluation (Q1–Q4) in LHL chocolates and HLL, particularly Q3. 

“Bitter” was dominant for all samples except HHH. The highest dominance rate was observed in sample LLL (0.47) and was dominant throughout the evaluation (Q1–Q4, 0.03–1.00). Bitter was also dominant throughout the evaluation for HLL. HLH (0.25) had the lowest bitter dominance rate and this attribute was dominant only towards the middle of the evaluation (Q2). Bitter was dominant in the first half of the evaluation (Q1–Q2) for HHL and dominant in intervals Q2–Q3 for LHL.

“Sweet” was dominant for samples with 43.6% sugar (HLH, HHL) and 51.5% sugar (HHH) at peak dominance rates of 0.31 (HHH) and 0.25 (HLH, HHL). It was dominant in intervals throughout the evaluation (0.09–1.00) for HHH, especially in Q4. Sweet was dominant in Q3 for HLH and was dominant at the beginning of evaluations (Q1–Q2) for HHL. 

The attribute “creamy” was dominant for samples with two or three H layers (HHH, HLH, HHL), or one H layer on the bottom (HLL), but only very briefly and with a low peak dominance rate (0.22 for all samples). The timepoint of dominance varied among these chocolate samples; for HHH towards the end of Q2, for HLH in the middle of Q2, for HHL in Q3, and for HLL in Q4. 

“Melting” was dominant for all samples with a peak dominance rate of 0.25 for samples with two or more L layers (LHL, HLL, LLL) or 0.28 for samples with two or more H layers (HHH, HLH, HHL). For HHH melting was dominant in Q2 and towards the end of Q3. For HLH melting was dominant during Q3, while for HHL it was dominant at the beginning of Q3 and throughout Q4. Melting was dominant in LHL at the end of Q4, briefly dominant in Q2 for HLL, and in Q4 for LLL. 

The TDS curves revealed that the sensory profiles of the 3D printed chocolates were influenced by the order in which H and L chocolate layers were arranged. Samples with the same amount of total sugar but a different layering order (e.g., HLH and HHL or LHL and HLL) had similar dominant attributes but differed in peak dominance rates and the time periods and duration that the attributes were dominant. 

### 3.4. TDS Difference Curves 

**Figure 2:**
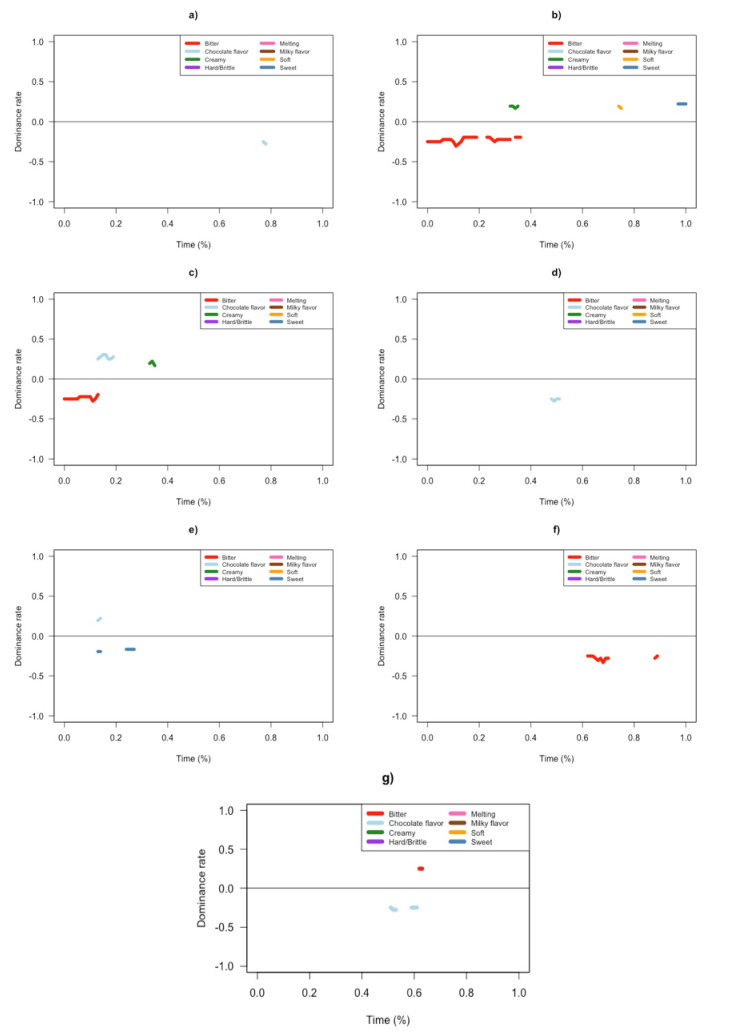
Temporal Dominance of Sensation (TDS) difference curves comparing samples with similar overall sweetness: (**a**) HHH-HLH; (**b**) HHH-HHL; (**c**) HLH-HHL; (**d**) HLH-LHL; (**e**) LHL-HHL; (**f**) LHL-LLL; (**g**) LLL-HLL.

TDS difference curves were plotted to highlight differences in dominance rates between samples with similar (Figure 2) and significantly different (Figure S2) mean sweetness intensity. 

For almost all of the samples with similar overall sweetness, differences were observed in the dominance rates for sweet, bitter, chocolate flavor, creamy and soft. The exception was between LHL and HLL, where no differences were observed. HLH was more dominant in chocolate flavor in Q3 compared to HHH (Figure 2a). HHH was more dominantly creamy in Q2, soft in Q3, and sweet in Q4 compared to HHL, while HHL was more dominantly bitter in Q1–Q2 (Figure 2b). HLH was more dominant in chocolate flavor in Q1 and creamy in Q2 compared to HHL, while HHL was more bitter in Q1 (Figure 2c). LHL was more dominant in chocolate flavor in Q2 compared to HLH (Figure 2d). HHL was more sweet in Q1 compared to LHL, while LHL was more dominant in chocolate flavor in Q1 (Figure 2e). LLL was more dominantly bitter in Q3 and Q4 compared to LHL (Figure 2f). LLL was more dominantly bitter in Q3, while HLL was more dominant in chocolate flavor in Q2 and Q3 (Figure 2g).

Samples perceived to be similar in overall sweetness intensity differed in their TDS profiles. The TDS difference curves suggested that other sensory attributes influenced overall sweetness perception. Furthermore, H and L chocolate layering order could have influenced the sensory profile of 3D printed chocolates, which could have affected overall sweetness. The 3D printed chocolate with an H layer in both the top and bottom (HLH) had the most similar temporal sensory profile to the sweetest control sample (HHH) (Figure 2a), while the sample with an H layer in the bottom only (HLL) had a profile that was most similar to the chocolate with the least amount of total % sugar (*w*/*w*) (LLL) (Figure 2g). 

Among samples with significantly different sweetness intensity, differences were observed in the dominance rates for sweet, bitter, chocolate flavor, and creamy. HHH was more dominantly sweet in Q1–Q2 and Q4 compared to LHL, while LHL was more dominantly bitter in Q2 and Q4 (Figure S2a). HLL was more dominantly bitter in Q1–Q2 and Q4 compared to HHH, while HHH was more dominantly sweet in Q1 and Q3–Q4 and creamy in Q2 and Q3 (Figure S2b). LLL was more dominantly bitter from Q1–Q4 compared to HHH, while HHH was more dominantly sweet in Q1–Q2 and Q4 (Figure S2c). HLL was more dominantly bitter in Q1 and Q4 and had a more dominant chocolate flavor in Q3 compared to HLH, while HLH was more dominantly sweet in Q1 and Q3 and creamy in Q2 (Figure S2d). LLL was more dominantly bitter in Q1 and Q3–Q4 compared to HLH, while HLH was more dominant in chocolate flavor in Q4 (Figure S2e). HLL was more dominant in chocolate flavor in Q1 and Q3, while HHL was more dominantly sweet in Q1 (Figure S2f). LLL was more dominantly bitter in Q3–Q4 compared to HHL, while HHL was more dominantly sweet in Q2 (Figure S2g). 

### 3.5. Principal Component Analysis (PCA)

**Figure 3:**
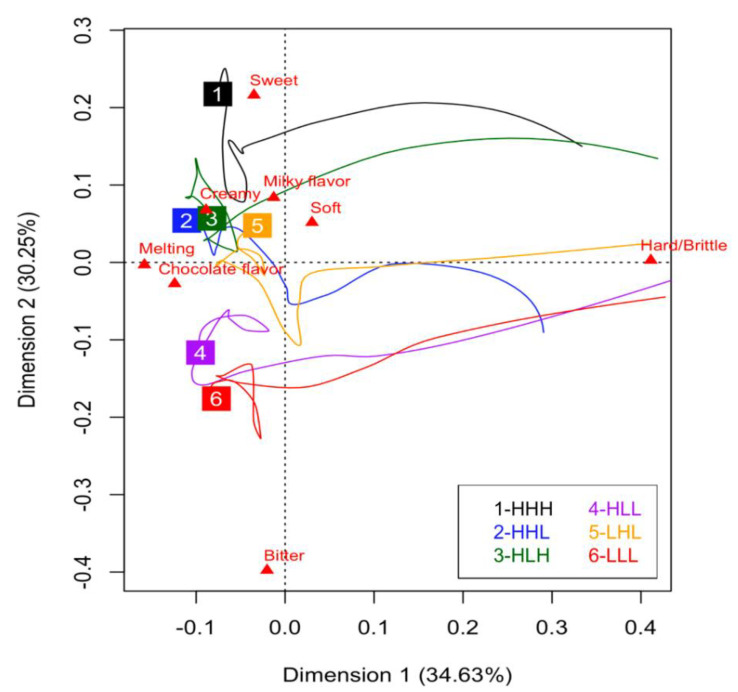
Principal Components Analysis (PCA) biplot representing the product trajectories of the six 3D printed chocolates during the TDS evaluation. Numbered squares represent trajectory end-points for the corresponding sample.

The first two dimensions explained 64.88% of the variance observed among the products (Figure 3). Dimension 1 was associated with the evaluation time points and the attributes “Hard/Brittle,” “Melting” and “Chocolate flavor” while dimension 2 was strongly associated with the opposing taste attributes “Bitter” and “Sweet.” “Chocolate flavor” was also associated with Dimension 3, which explained 16.17% of the product variation (data not shown).

For all six 3D printed chocolates, the first perceived dominant attribute was “Hard/Brittle”, and the last perceived dominant attributes were “Chocolate flavor” and “Melting,” as evidenced by dimension 1. Three groupings were defined by Dimension 2. HHH was its own group and was most associated with sweet. The sweetness dominance of HHL, HLH, and LHL was perceived similarly, while the group of HLL and LLL was more associated with bitter compared to the other two groups. Dimension 1 indicated that the similarities in sweetness within the three groups occurred towards the end of the evaluation. For the HLL and LLL group, in the middle of the evaluation, HLL was more dominant in sweet while LLL was more dominant in bitter. For the grouping of HHL, HLH and LHL, in the middle of the evaluation, HHL and LHL had a similar trajectory, but HLH had a trajectory more similar to HHH.

## 4. Discussion

In this study, three-layered sugar-reduced 3D printed chocolates were generated to taste as sweet as samples with 19% greater total sugar content. The position of H and L chocolate layers influenced the temporal sensory profile and perceived overall sweetness of the chocolate samples. Overall, the 3D printed chocolates were well-liked, and liking was not affected by temporal sensory attribute changes. 

Layering chocolates with different amounts of sugar achieved a 19% reduction in sugar without a reduction in overall sweetness perception. However, a 32% sugar reduction in sweetness was detected by participants. These results are similar to other studies of layered tastants including sugar in gels [5], in which a large tastant concentration gradient between the layers creates a contrast effect. By alternating concentrations of sucrose in layers, taste receptors are stimulated in intervals with either low or high sugar concentrations, causing a sweetness enhancement effect. 

Similar results were observed for liquid solutions, where sweetness was perceived to be more intense in an alternating presentation of high and low sucrose solutions in short intervals [28]. In this method, a conscious perception of the contrast was not required for taste enhancement. The contrast effect may also reduce adaptation, the gradual decrease in taste receptor response with continuous stimulation [29]. In the present research, the sample with contrasting layers LHL (34.9% sugar) was perceived to be similar in sweetness to samples with 41.6% sugar (HHL and HLH), while HLL (34.0% sugar) was not. Discontinuous taste receptor stimulation may be responsible for this. 

The layering order of H and L chocolates affected the temporal sensory profile of the 3D printed chocolates. Differences were observed in the peak dominance rates, time periods, and duration of the dominant attributes “Bitter,” “Chocolate flavor,” and “Creamy” for 3D printed chocolates with the same amount of total sugar but different layering order (HLH and HHL). Furthermore, these changes to the temporal sensory profile appeared to affect perceived sweetness. When the H layer was in the bottom only (HLL), or not present at all (LLL), “Bitter” was dominant compared to HHH, which contributed to significantly decreased perceived overall sweetness. Placement of H chocolate in the middle layer only (LHL, 34.9% sugar), resulted in a temporal sensory attribute profile most similar to HLH and HHL (41.6% sugar), with perceived similar sweetness. A temporal sensory profile and sweetness similar to HHH chocolate (highest total % sugar (*w*/*w*)) was created by placing the H chocolate in both the top and bottom layers (HLH). Evaluation of layering sequences not evaluated in this study (LLH, LHH) could provide more insight on specific influences of H layer placement.

The hypothesis that the placement of sweeter chocolate at bottom layers would increase the overall sweetness perception of layered chocolates was confirmed. Samples with 43.56% sugar (HLH, HHL) were comparable in sweetness to a conventional 51.52% sugar chocolate (HHH). Similarly, cream-based snacks with a 35% reduction in salt and salt-associated aroma were found to be saltier than the reference snack when the salty aroma layer was at the bottom and the saltier layer was at the top [8]. In contrast, sweetness intensity was not different among four four-layered gels with different layering arrangements and an overall 10% (*w*/*w*) sugar concentration [5]. In the present research, LHL (35.3% sugar) did not have a high sugar layer at the bottom but tasted similar in sweetness to HLH and HHL (43.56% sugar). This suggested an influence of factors in addition to layering order. 

Perceived dominance of some sensory attributes and timing of the dominance perception during consumption may have affected overall sweetness perception. Significantly higher dominance rates for “creamy” or “sweet” appeared to increase sweetness perception. These attributes are more strongly associated with milk chocolate [30], which is generally sweeter than dark chocolate due to lower amounts of cocoa and higher amounts of sugar [31]. Higher dominance rates of attributes associated with dark chocolates, such as “bitter” or “chocolate flavor” [31], are associated with decreased sweetness perception. Dominance time for “bitter” appeared to influence perceived overall sweetness due to primacy and recency effects [32]. Greater dominance of “bitter” in the first half of the evaluation or near the end of the evaluation decreased the perceived overall sweetness intensity for that chocolate. 

For each 3D printed chocolate, each sensory attribute was dominant at some point during the evaluation with the exception of “Milky flavor.” Based on previous TDS studies of milk chocolate or chocolate with cocoa content similar to the chocolate evaluated in this study (41%, 53%, or 55%), H chocolate (47% cocoa) was expected to be dominant in the attributes “Hard,” “Brittle,” “Sweet,” “Melting,” “Soft,” “Cocoa,” “Milk,” and “Creaminess” [19,20,21]. L chocolate (72% cocoa) was expected to have similar dominant attributes to dark chocolates with 63% or 70% cocoa, described as dominant in “Crunchy,” “Cocoa”, “Bitter” and “Melting” [18,20]. As expected, “Bitter” was dominant for all samples with at least one L layer, while “Sweet” was dominant for samples with two or three H layers and a higher total % sugar (41.6% and 51.5%). “Creamy” was associated with H chocolate, as it was only dominant for samples with two or three H layers.

The HLL chocolate may have been perceived as “Bittersweet” by the participants. Both “Bitter” and “Sweet” were dominant in the first half of the evaluation, but during the dominance periods, the dominance rate for “Sweet” was lower than for “Bitter”. Bittersweet was excluded from the TDS attributes list provided to the participants to avoid confusion when selecting a single taste attribute as dominant. The dominance of “Hard/Brittle” at the beginning of evaluations was likely related to initial chewing. The hollow shape and thin walls (4.37mm) of the printed chocolates could have contributed to this perception. 

The dominance rates, number of dominant attributes selected by participants, and number of responses collected in this research were comparable to previous temporal studies on milk and dark chocolate and chocolate products. In previous studies, dominance rates between 0.2–0.6 for chocolate attributes were observed, and some attributes were not dominant at any point during TDS evaluations [15,20,33,34]. Higher dominance rates (>0.8) were recorded when there were only a few attributes to select from [17] or for added attributes, such as “fruity” for orange dark chocolate [18]. Since TDS evaluates dominant attributes over time, the sub-threshold perception could result in non-dominant attributes. 

The 3D printed chocolates were well-liked by participants, as regular consumption of both milk and dark chocolate was a participation criterion. The differences observed among the TDS profiles of similarly sweet samples did not affect the overall liking of the 3D printed chocolates. To identify temporal sensory attributes as drivers of liking, future studies could use a technique such as a penalty lift analysis to link specific temporal attributes to liking scores among chocolates with more variable sensory and quality attributes [19]. A larger number of panel participants to permit stratification based on chocolate preference and consumption would facilitate further insights into the TDS profile of the chocolate samples. 

To more clearly elucidate the effect of layering order, future larger studies could include all possible variations of the order of the three layers in the 3D printed chocolates, samples with a greater number of layers, or greater concentration gradient between the layers. Furthermore, chocolates with discrete high sugar regions at targeted locations anywhere within the three-dimensional space could also be studied. Three-dimensional food printing technology is progressing rapidly, and further sugar reduction could be achieved with refined research methods. There is a motivation for the food industry to continue research on sugar reduction strategies to achieve specific sugar reduction targets [35,36]. Furthermore, the increasing popularity of Ketogenic, Mediterranean, and Paleo diets [37] has promoted reduced consumption of both carbohydrates and high sugar processed foods. 

This study evaluated the temporal dominance of sensations of non-sugar-reduced and sugar-reduced chocolates created by the layering of high and low sugar chocolate using a 3D printer. This sugar reduction method influenced temporal sensory attributes, which along with layer positioning and the tastant contrast effect, contributed to enhancing sweetness intensity. The 3D printed chocolates were well-liked and temporal sensory profile changes did not negatively affect overall liking.

## 5. Conclusions

The layering of high and low sugar chocolate by 3D printing achieved up to a 19% sugar reduction without changes in overall sweetness perception and overall liking. A discontinuous stimulation of the taste receptors with a sufficient sugar concentration gradient between the layers contributed to this sweetness enhancement. The layering order from the bottom to the top of alternating high and low concentration sugar layers changed the temporal sensory attribute profile of 3D printed chocolates, but these changes did not influence overall liking. Future studies could utilize 3D printing to generate chocolates with varying numbers of layers, different concentration gradients between the layers, and all possible layering orders to more clearly describe the effect of these variables on perceived temporal sensory profile and overall sweetness. Three-dimensional food printing technology is progressing rapidly and further sugar reduction could be achieved with refined research methods.

## Figures and Tables

**Table 1:** Sensory attribute definition list for Temporal Dominance of Sensation (TDS) evaluations.

Attribute ^1^	Type of Attribute	Definition	Examples
Sweet	Taste	The taste of table sugar	Table sugar
Bitter	Taste	A sharp, pungent taste	Dark chocolate, strong black coffee, tonic water, or aspirin
Milky flavor	Taste	Reminiscent of the taste of fresh milk or the characteristic dairy note of milk chocolate	Fresh milk, milk chocolate
Chocolate flavor	Taste	The characteristic flavor of chocolate products	Cocoa powder, hot chocolate, chocolate milk, Hershey’s kisses, Cadbury Dairy Milk Chocolate bars
Creamy	Texture	The fatty mouthfeel or thick coating on the tongue	Milkshake, heavy cream
Melting	Texture	A change in the chocolate from a solid to liquid	Ice cream melting in the mouth
Hard/ Brittle	Texture	High resistance to pressure from front teeth when biting through the sample, and breaks or shatters into crumbs or pieces	Shortbread, vanilla wafer, peanut brittle, gingersnap cookies

^1^ Soft was included in the list of attributes for the TDS evaluation; a definition was not presented in the sensory attribute definition list.

**Table 2:** Demographic and product use results of the consumer panelists (*n* = 72).

	Category	Number (% Frequency)
Sex	Male	24 (33)
	Female	48 (67)
Age	18–25 years	42 (58)
	26–35 years	19 (26)
	Greater than 36 years	11 (16)
Preference for sweet or savory food	Savory food	15 (21)
	Sweet food	23 (32)
	No preference	34 (47)
Preference for milk or dark chocolate	Milk chocolate	39 (54)
	Dark chocolate	33 (46)
Type of chocolate usually consumed	White chocolate	2 (3)
	Milk chocolate	26 (36)
	Dark chocolate	21 (29)
	Chocolate with fruit, nuts or other flavorings (e.g., almonds, salt, orange)	13 (18)
	Chocolate as part of a mix of multiple ingredients (e.g., O’Henry, Mars, Coffee Crisp, Crispy Crunch)	10 (14)
Consumption frequency for usual type of chocolate	Daily	8 (11)
	3–4 times per week	24 (33)
	Once per week	30 (42)
	Once per month	7 (10)
	A few times per year	3 (4)

**Table 3:** Total % sugar (*w*/*w*) and mean sweetness and liking ± standard deviation ^1^ for the 3D printed chocolates (*n* = 72).

Sample ^2^	Total % Sugar	Sweetness ^3^	Liking ^4^
HHH	51.5	3.3 ^a^ ± 0.8	6.6 ± 1.5
HLH	41.6	3.2 ^ab^ ± 0.7	6.9 ± 1.1
HHL	41.6	2.9 ^ab^ ± 0.9	6.5 ± 1.5
LHL	34.9	2.7 ^bc^ ± 0.8	6.6 ± 1.4
HLL	34.0	2.4 ^c^ ± 0.9	6.8 ± 1.4
LLL	26.7	2.3 ^c^ ± 1.0	6.4 ± 1.6

^1^ Samples with different lower case superscripted letters within a column are significantly different (*p* ≤ 0.05). ^2^ Upper case letters represent the layering order from bottom to top (H: 51.5%, L: 26.7% total sugar). Participants were instructed to place the bottom layer on the tongue for evaluations. ^3^ Samples were evaluated on a 5-point scale (1 = not at all sweet, 5 = extremely sweet). ^4^ Samples were evaluated on a 9-point scale (1 = dislike extremely, 9 = like extremely).

## Data Availability

The data presented in this study are available on request from the corresponding author. The data are not publicly available due to ethical reasons.

## Supplementary Materials

The following are available online at https://www.mdpi.com/article/10.3390/foods10092082/s1, Figure S1. TDS curves for each 3D printed chocolate sample: (a) HHH (b) HLH (c) HHL (d) LHL (e) HLL (f) LLL. Colored lines represent: Bitter, Chocolate flavor, Melting, Milky flavor, Creamy, Soft, Hard/Brittle, Sweet. Figure S2. TDS difference curves comparing samples with significantly different sweetness: (a) HHH-LHL (b) HHH-HLL (c) HHH-LLL (d) HLH-HLL (e) HLH-LLL (f) HHL-HLL (g) HHL-LLL.
