# Supplementary material for: Temporal Sensory Perceptions of Sugar-Reduced 3D Printed Chocolates
Source: Foods. 2021 Sep 3;10(9):2082. doi: 10.3390/foods10092082 (PMC8465193; doi:10.3390/foods10092082)
Supplement: Supplementary file 1 [file foods-10-02082-s001.zip › foods-1312736-supplementary.pdf]

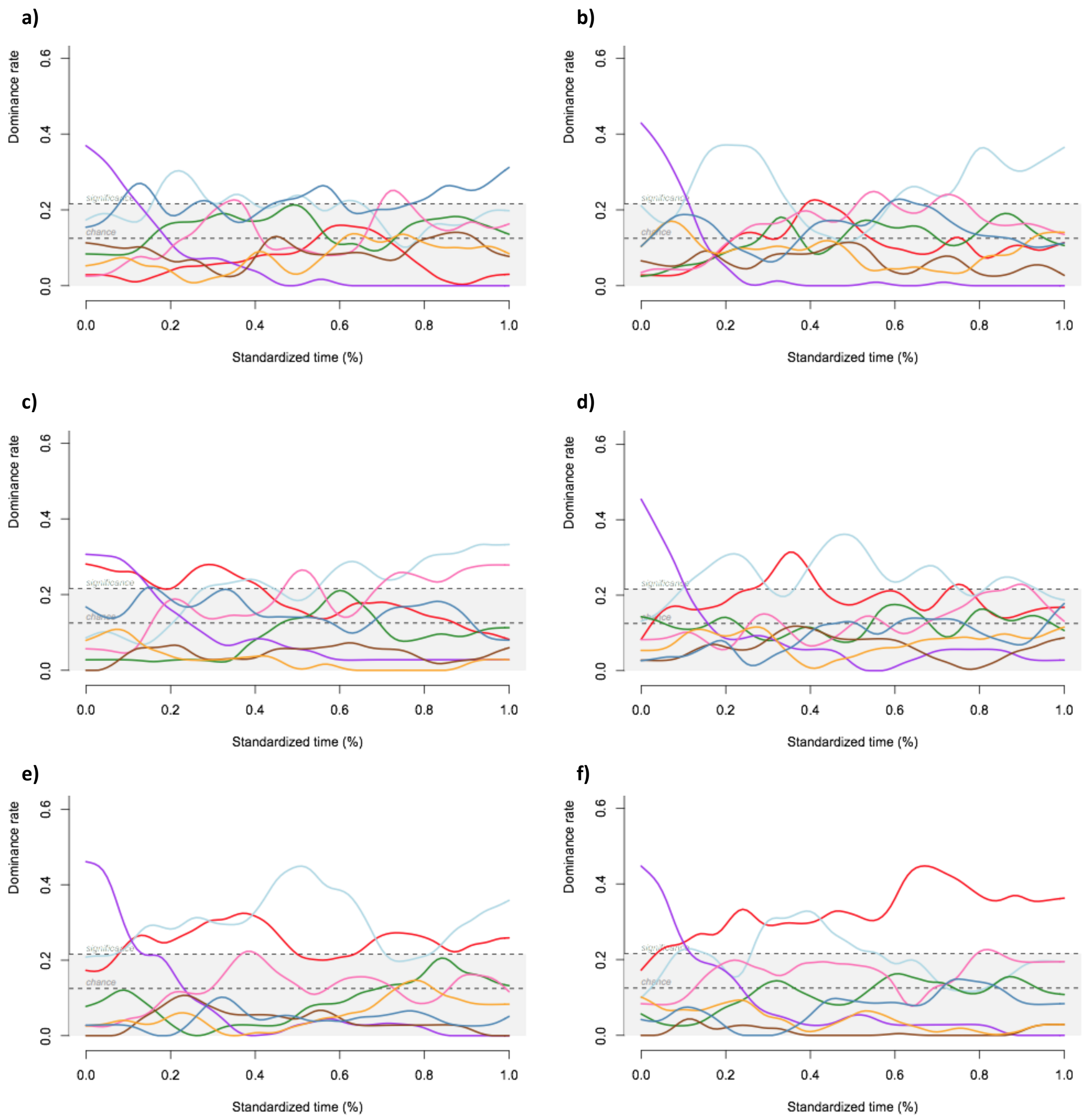

Figure S1. TDS curves for each 3D printed chocolate sample: **a)** HHH **b)** HLH **c)** HHL **d)** LHL **e)** HLL **f)** LLL. Colored lines represent: Bitter, Chocolate flavor, Melting, Milky flavor, Creamy, Soft, Hard/Brittle, Sweet.

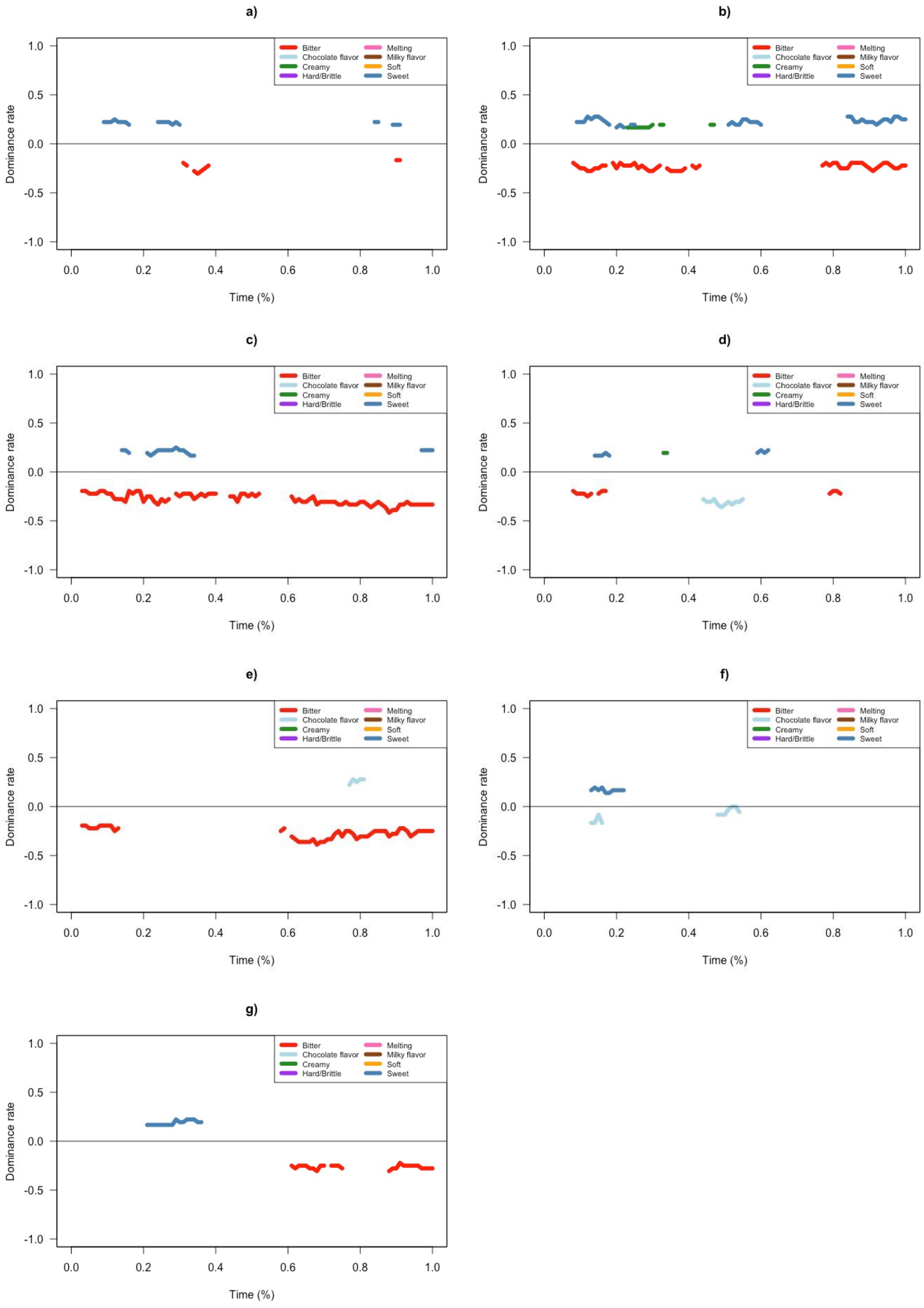

Figure S2. TDS difference curves comparing samples with significantly different sweetness: **a)** HHH-LHL **b)** HHH-HLL **c)** HHH-LLL **d)** HLH-HLL **e)** HLH-LLL **f)** HHL-HLL **g)** HHL-LLL
